# The combined impact of smoking, obesity and alcohol on life-expectancy trends in Europe

**DOI:** 10.1093/ije/dyaa273

**Published:** 2021-01-11

**Authors:** Fanny Janssen, Sergi Trias-Llimós, Anton E Kunst

**Affiliations:** 1 Netherlands Interdisciplinary Demographic Institute—KNAW/University of Groningen, The Hague, The Netherlands; 2 Population Research Centre, Faculty of Spatial Sciences, University of Groningen, The Netherlands; 3 Department of Non-communicable Disease Epidemiology, Faculty of Epidemiology and Population Health, London School of Hygiene & Tropical Medicine, London, UK; 4 Center for Demographic Studies, Centres de Recerca de Catalunya (CERCA), Bellaterra, Spain; 5 Department of Public and Occupational Health, Amsterdam UMC, University of Amsterdam, Amsterdam, The Netherlands

**Keywords:** Health behaviour, lifestyle, Europe, life expectancy, mortality, time trends

## Abstract

**Background:**

Smoking, obesity and alcohol abuse greatly affect mortality and exhibit a distinct time dynamic, with their prevalence and associated mortality rates increasing and (eventually) declining over time. Their combined impact on secular trends in life expectancy is unknown but is relevant for understanding these trends. We therefore estimate the combined impact of smoking, obesity and alcohol on life-expectancy trends in Europe.

**Methods:**

We used estimated national age-specific smoking-, obesity- and alcohol-attributable mortality fractions for 30 European countries by sex, 1990–2014, which we aggregated multiplicatively to obtain lifestyle-attributable mortality. We estimated potential gains in life expectancy by eliminating lifestyle-attributable mortality and compared past trends in life expectancy at birth (e0) with and without lifestyle-attributable mortality. We examined all countries combined, by region and individually.

**Results:**

Among men, the combined impact of smoking, obesity and alcohol on e0 declined from 6.6 years in 1990 to 5.8 years in 2014, mainly due to declining smoking-attributable mortality. Among women, the combined impact increased from 1.9 to 2.3 years due to mortality increases in all three lifestyle-related factors. The observed increase in e0 over the 1990–2014 period was 5.0 years for men and 4.0 years for women. After excluding lifestyle-attributable mortality, this increase would have been 4.2–4.3 years for both men and women.

**Conclusion:**

Without the combined impact of smoking, obesity and alcohol, the increase over time in life expectancy at birth would have been smaller among men but larger among women, resulting in a stable increase in e0, parallel for men and women.

**Key Messages**

- We observed a large combined impact of smoking, obesity, and alcohol on trends in life expectancy at birth (e0) in Europe. Without its impact, the increase over time in e0 would have been smaller among men but larger among women.
- We identified a stable increase in life expectancy for non-lifestyle-attributable mortality, which occurred in parallel for men and women in most European countries, and was, for men, more similar between countries than for the observed life-expectancy trends.
- Distinguishing between non-lifestyle-attributable mortality and lifestyle-attributable mortality is important to better understand current trends in life expectancy and to predict future trends.

## Introduction

Smoking, alcohol abuse and behavioural factors resulting in overweight and obesity (unhealthy diets and insufficient physical activity) are important contributors to non-communicable-disease mortality.^1^ Smoking, alcohol and obesity are considered to be three key preventable risk factors and public-health problems in Europe,^2^^,^^3^ with a high impact on mortality and life expectancy.^4–14^ However, the contributions of smoking, obesity and alcohol are not constant over time, because these lifestyle factors tend to evolve as wave-shaped lifestyle ‘epidemics’ characterized by an initial unprecedented increase followed (eventually) by a decline.^15–20^ Given important (recent) stagnations in increases in life expectancy in Europe over time,^21–25^ it is important to assess how these lifestyle factors influenced secular trends in life expectancy in different European countries.

Previous research clearly revealed the time-varying nature of smoking-, obesity- and alcohol-attributable mortality. Past trends in smoking-attributable mortality show a clear wave pattern of increases followed by declines that occurred approximately 30–40 years later than a similar wave pattern for smoking prevalence.^15^ This wave pattern has been observed among men in all European countries, whereas smoking-attributable mortality appears to still be increasing among women in the majority of European countries.^16^ The tripling of obesity prevalence in Europe since 1980^26^ has resulted in clear increases in obesity-attributable mortality fractions over the 1975–2016 period,^27^ albeit with a recent slowing-down of the rates of increase.^28^ Alcohol prevalence and, subsequently, alcohol-attributable mortality levels, which are especially high among Eastern-European men,^29^^,^^30^ had been mostly increasing in Eastern and North-western Europe, although recently they have been stagnating or declining in most of these countries. In most South-western-European countries, these levels have been steadily declining, albeit more slowly in recent years.^19^^,^^20^

Also, various Global Burden of Disease studies estimated trends in risk-attributable deaths and Disability Adjusted Life Years for different (groups of) risks (behavioural, environmental, occupational, metabolic) from 1990 onwards.^31–34^ We will complement this work by estimating the effects of lifestyle-attributable mortality on secular trends in life expectancy.

Previous studies on the impact of smoking-, obesity- and alcohol-attributable mortality on trends in life expectancy mostly examined the impact for a single lifestyle factor and usually for only a selection of countries.^22^^,^^23^^,^^27^^,^^30^^,^^35–37^ They found that smoking played an important role in the stagnation of life-expectancy increases among men in many North-western European countries in the 1950s and 1960s, and in other European countries and among women in more recent decades.^22^^,^^23^^,^^35^ For obesity, an increasingly large effect on life-expectancy levels over recent decades was observed.^27^ A similar trend was found for alcohol in Finland.^36^ In addition, the dissimilar trends in alcohol prevalence between Eastern and non-Eastern Europe have greatly contributed to diverging (1990–2005) and converging (2005 onwards) life-expectancy levels across Europe.^30^ Although these previous results suggest that the combined effect of smoking, obesity and alcohol on life-expectancy trends could be large, this combined effect has not yet been studied.

Our objective is, therefore, to estimate, for the first time, the combined impact of smoking, obesity (BMI ≥ 30 kg/m^2^) and alcohol abuse on trends in life expectancy for men and women in Europe and to determine how trends in life expectancy would look like without this combined impact. We study the majority of European countries (*N* = 30) combined, with additional attention on differences by region (North, West, South, Central, East) and country.

## Data and methods

For the 30 countries under study, we used previously calculated estimates of smoking-, obesity- and alcohol-attributable mortality fractions by sex and single adult ages (smoking: 35–100 years; obesity and alcohol: 20–100 years) (see next paragraph).^16^^,^^20^^,^^38^ In addition, we used age-, sex-, country- and year-specific all-cause mortality and population data from the Human Mortality Database.^39^ We studied the period 1990–2014 because alcohol-attributable mortality could be estimated only since 1990 and smoking-attributable mortality could not be estimated after 2014 for several countries due to the unavailability of lung-cancer-mortality rates for more recent years (see Supplementary Data & Methods—Appendix 1, available as Supplementary data at *IJE* online, for the data availability by country for the different elements).

Smoking-attributable-mortality fractions were indirectly estimated using a simplified version of the commonly applied Peto-Lopez methodology.^16^^,^^40^^,^^41^ The method estimates exposure to smoking based on lung-cancer mortality rates that are adjusted for the part not due to smoking and applies to this exposure the relative risks (RRs) of dying from smoking. For obesity-attributable mortality, the estimates stem from the application of the population-attributable fraction formula to (estimated) prevalence data^42^ using all-cause RRs of dying from obesity,^43^ in line with previous studies, e.g.^27^. Alcohol-attributable mortality fractions were estimated based on alcohol-attributable mortality rates obtained from the Global Burden of Disease 2017 study,^31^^,^^44^ adjusted for ages 65+ using the age pattern for alcohol-related causes of death.^20^

To estimate the share of mortality due to smoking, obesity and alcohol combined, we used the multiplicative aggregation of the fractions for the individual risk factors, using the formula^45^:

$${\mathrm{LAMF}=\;PAF}_{1..n}=1-\;\prod_{i=1}^{n}{(1-PAF}_{i})$$

where *i* stands for the individual risk factor and ${\mathrm{PAF}}_{1.n}\;$stands for the lifestyle-attributable mortality fraction for the three lifestyle factors combined, which we refer to as the lifestyle-attributable mortality fraction (LAMF). This aggregation rests on the strong assumption that risk factors are independent and uncorrelated.^45^ However, validation studies have demonstrated that the resulting estimates may closely resemble the true combined effect.^31^^,^^46^

By multiplying the age- and sex-specific smoking-, obesity-, alcohol- and lifestyle-attributable mortality fractions to the respective all-cause mortality rates, we obtained age- and sex-specific smoking-, obesity-, alcohol- and lifestyle-attributable mortality rates. By multiplying the all-cause mortality rates by one minus the fractions, we obtained the age- and sex-specific non-smoking-, non-obesity-, non-alcohol- and non-lifestyle-attributable mortality rates.

To aid the interpretation of the results from our main analysis (see below), we performed two initial analyses. First, we examined the trends over time in the share of mortality that is attributable to the different lifestyle factors. We obtained shares across adult ages (20–100 years) by applying direct standardization to the age-specific fractions using the age composition of deaths for the specific populations in 2010. Second, we examined the impact on life expectancy at birth (e0) of the separate and combined lifestyle factors, and the trends therein over time, by calculating the potential gain in life expectancy (PGLE)^47^ if smoking-, obesity-, alcohol- and lifestyle-attributable mortality were eliminated. The PGLE values are calculated by comparing the e0 value for all-cause mortality to the e0 value based on life-table calculations applied to non-smoking-, non-obesity-, non-alcohol- and non-lifestyle-attributable mortality rates, respectively.

For our main aim of estimating the (combined) impact of smoking, obesity and alcohol on life-expectancy trends in Europe, we graphically compared the trends over time in observed e0 (= for all-cause mortality) with the trends in estimated e0 values (= for non-smoking-, non-obesity-, non-alcohol- and non-lifestyle-attributable mortality) (see before). To compare the overall change in observed e0 over the 1990–2014 period with this change in estimated e0 values, we subtracted the respective e0 values in 1990 from the respective e0 values in 2014.

See the Supplementary Data & Methods document, available as Supplementary data at *IJE* online, for more details.

## Results

**Figure 1:**
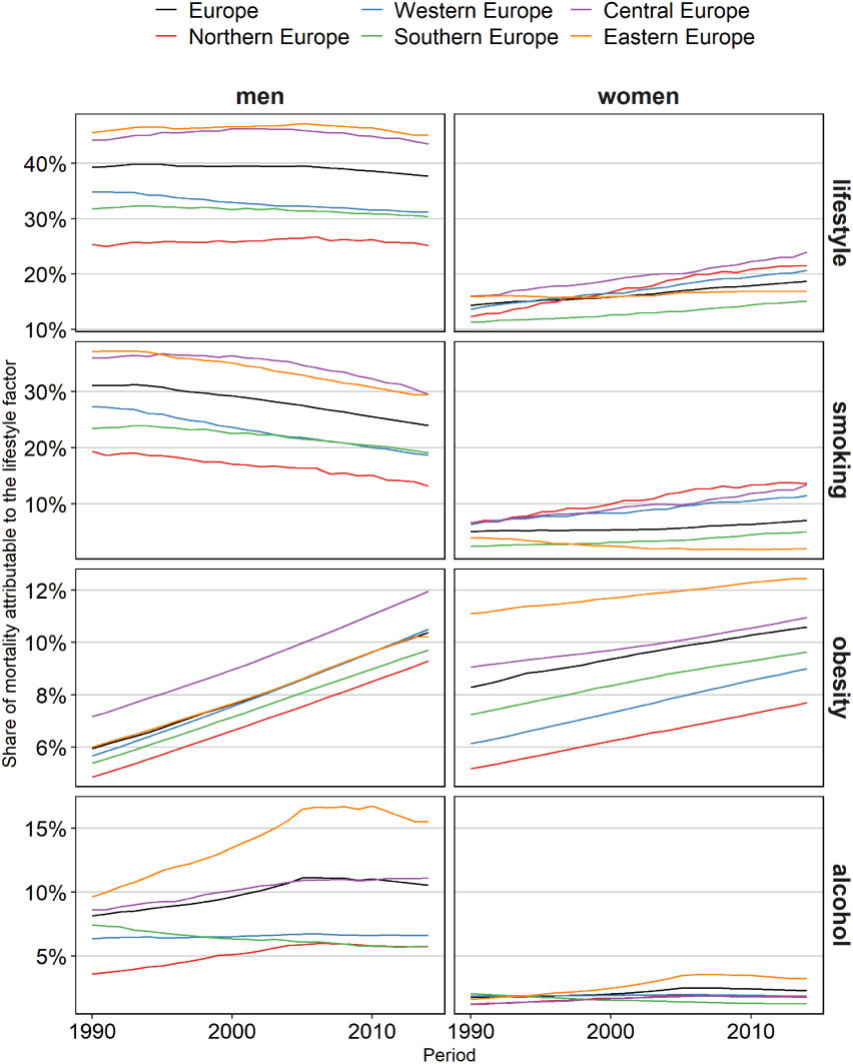
Trends over time in age-standardized smoking-, obesity- and alcohol-attributable mortality fractions (separately and combined) (%), ages 20–100 years, 1990–2014*, for the different European regions, by sex. Lifestyle refers to smoking, obesity and alcohol combined. *For 2011 up to 2014, the weighted averages were calculated using the data for the latest available year: Bulgaria (2010), Greece (2013), Ukraine (2012) and Russia (2013).

**Figure 2:**
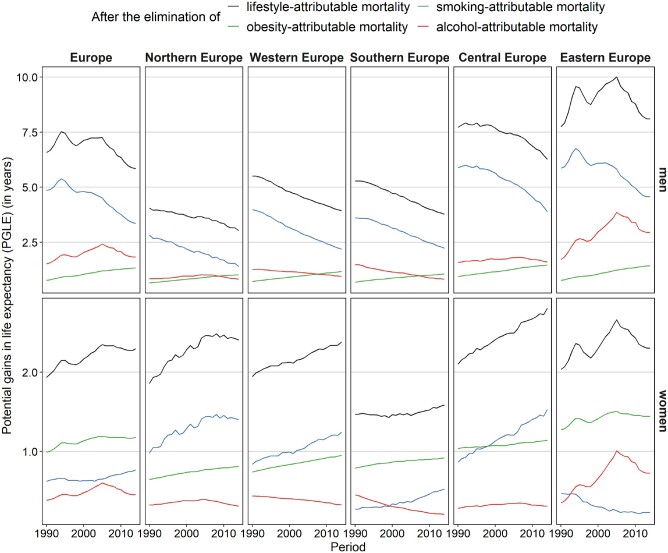
Trends over time in the potential gain in life expectancy (PGLE) after eliminating smoking-, obesity- and alcohol-attributable mortality (separately and combined), 1990–2014*, by European region and sex. Lifestyle-attributable mortality refers to mortality that is attributable to smoking, obesity and alcohol combined. *For 2011 up to 2014, the weighted averages were calculated using the data for the latest available year: Bulgaria (2010), Greece (2013), Ukraine (2012) and Russia (2013).

**Figure 3:**
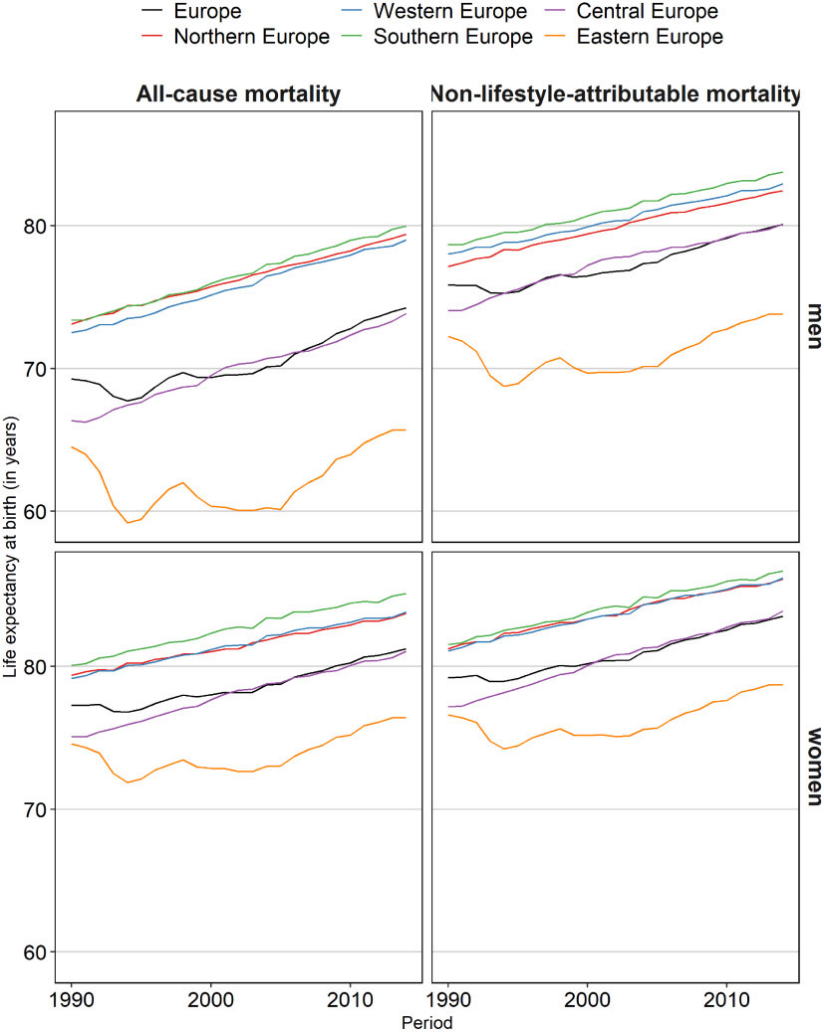
Trends in life expectancy at birth (e0), observed (= all-cause mortality) versus after excluding lifestyle-attributable mortality (= non-lifestyle-attributable mortality), 1990–2014*, by European region and sex. Lifestyle-attributable mortality refers to mortality that is attributable to smoking, obesity and alcohol combined. *For 2011 up to 2014, the weighted averages were calculated using the data for the latest available year: Bulgaria (2010), Greece (2013), Ukraine (2012) and Russia (2013).

Before describing the results of our main analysis in Table 2 and Figure 3, we first describe the results of our background analyses in Figure 1, Table 1 and Figure 2. Whereas the tables and figures in the manuscript show the results by European region, the respective supplementary tables and figures, available as Supplementary data at *IJE* online, show the country-specific results.

**Table 1:** Impact of smoking, obesity and alcohol (separately and combined) on life expectancy at birth (e0) in 30 European countries, by sex and region, 1990 and 2014^a^ by means of estimates of the potential gain in life expectancy (PGLE) (in years) from the elimination of mortality linked to the respective lifestyle factor. Lifestyle refers to smoking, obesity and alcohol combined

		PGLE 1990 (in years)					PGLE 2014^a^ (in years)			
	e0 1990	Lifestyle	Smoking	Obesity	Alcohol	e0 2014^a^	Lifestyle	Smoking	Obesity	Alcohol
**Men**										
Europe	69.28	6.58	4.85	0.78	1.52	74.26	5.84	3.36	1.34	1.83
Northern Europe	73.11	4.06	2.83	0.66	0.84	79.42	3.04	1.40	1.03	0.83
Western Europe	72.51	5.51	3.98	0.72	1.27	79.02	3.94	2.19	1.18	0.96
Southern Europe	73.40	5.28	3.61	0.69	1.48	79.99	3.78	2.23	1.06	0.83
Central Europe	66.34	7.72	5.89	0.94	1.59	73.86	6.27	3.88	1.47	1.61
Eastern Europe	64.52	7.74	5.87	0.77	1.72	65.72	8.10	4.56	1.43	2.95
**Women**										
Europe	77.28	1.94	0.62	0.99	0.38	81.23	2.30	0.77	1.18	0.45
Northern Europe	79.38	1.86	0.97	0.64	0.32	83.72	2.41	1.40	0.81	0.31
Western Europe	79.16	1.95	0.84	0.74	0.43	83.82	2.38	1.24	0.95	0.32
Southern Europe	80.08	1.47	0.27	0.79	0.45	85.10	1.59	0.52	0.92	0.20
Central Europe	75.07	2.10	0.86	1.03	0.28	81.08	2.81	1.53	1.14	0.31
Eastern Europe	74.57	2.04	0.47	1.27	0.35	76.44	2.31	0.23	1.44	0.72

a For 2014, the weighted averages were calculated using the data for the latest available year for those countries for which 2014 data were missing: Bulgaria (2010), Greece (2013), Ukraine (2012) and Russia (2013).

Northern Europe: Denmark, Finland, Iceland, Norway, Sweden.

Western Europe: Austria, Belgium, Germany, France, Ireland, Luxembourg, Netherlands, Switzerland, the UK.

Southern Europe: Greece, Italy, Portugal, Spain.

Central Europe: Bulgaria, Czech Republic, Hungary, Poland, Slovakia, Slovenia.

Eastern Europe: Belarus, Estonia, Latvia, Lithuania, Ukraine, Russia.

**Table 2:** Impact of smoking, obesity and alcohol (separately and combined) on the change in life expectancy at birth (e0) in 30 European countries from 1990 until 2014^a ^by sex and region

	Change in e_0_ 1990–2014^a^ (in years)				
	Observed	Without smoking	Without obesity	Without alcohol	Without smoking, obesity and alcohol
**Men**					
Europe	4.98	3.48	5.54	5.29	4.24
Northern Europe	6.31	4.89	6.69	6.30	5.29
Western Europe	6.51	4.72	6.96	6.20	4.93
Southern Europe	6.59	5.22	6.96	5.94	5.09
Central Europe	7.52	5.51	8.04	7.54	6.06
Eastern Europe	1.20	–0.10	1.85	2.42	1.56
**Women**					
Europe	3.96	4.10	4.15	4.03	4.32
Northern Europe	4.34	4.77	4.50	4.33	4.89
Western Europe	4.66	5.07	4.87	4.55	5.10
Southern Europe	5.03	5.28	5.16	4.78	5.14
Central Europe	6.01	6.68	6.11	6.03	6.71
Eastern Europe	1.87	1.63	2.04	2.24	2.14

a For countries for which 2014 data were missing, we used data up until the latest available year: Bulgaria (2010), Greece (2013), Ukraine (2012) and Russia (2013).

Across all 30 European countries in 2014, the age-standardized share of mortality due to smoking, obesity and alcohol combined (= lifestyle-attributable mortality fraction) was 38% among men and 19% among women. Among men, the LAMF mostly declined over the 1990–2014 period, albeit only recently in many Northern European and Central and Eastern European (CEE) countries (Figure 1 and Supplementary Figure 1, available as Supplementary data at *IJE* online). Among women, the LAMF generally increased over time. The declines in the LAMF for men were mostly driven by declines in smoking-attributable mortality, which started later in CEE. These declines in smoking-attributable mortality were, however, counterbalanced by increases in obesity-attributable mortality and, in Northern and CEE countries, as well by (decelerating) increases in alcohol-attributable mortality. The increases in the LAMF for women were driven by increases in smoking-attributable mortality, in obesity-attributable mortality and—particularly in CEE—in alcohol-attributable mortality.

The potential gain in life expectancy (PGLE) by eliminating lifestyle-attributable mortality declined from 6.6 to 5.8 years among men and increased from 1.9 to 2.3 years among women across the 30 European countries over the 1990–2014 period (Table 1 and Figure 2). Among men in Eastern Europe, the combined impact of smoking, obesity and alcohol on life expectancy was the greatest and was—in contrast to other regions—increasing up to 2005 primarily due to the increases in the PGLE for alcohol (Table 1 and Supplementary Table 1, available as Supplementary data at *IJE* online, Figure 2 and Supplementary Figure 2, available as Supplementary data at *IJE* online). Among women, the impact was the smallest and hardly changing in Southern Europe because moderate increases in PGLE for smoking and obesity were almost offset by small declines in PGLE for alcohol.

Between 1990 and 2014, e0 increased by 5.0 years among men across the 30 European countries (Table 2). Without lifestyle-attributable mortality, this increase would have been 4.2 years (i.e. 0.8 fewer years). The impact of lifestyle factors on life-expectancy trends was largest among men in Western, Southern and Central Europe, where the increase in e0 would have been about 1.5 years lower without lifestyle-attributable mortality (Table 2 and Supplementary Table 2, available as Supplementary data at *IJE* online). By contrast, among Eastern-European men, excluding lifestyle-attributable mortality would have resulted in a 0.4-year higher increase in e0. Among European women, the average increase in e0 from 1990 to 2014 was 4.0 years, whereas it would have been 4.3 years (i.e. 0.3 years higher) after excluding lifestyle-attributable mortality (Table 2). An increase in e0 after excluding lifestyle-attributable mortality can be observed for women in all European regions and countries (Table 2 and Supplementary Table 2, available as Supplementary data at *IJE* online). This impact of lifestyle factors on life-expectancy trends was biggest in Central Europe (difference of 0.7 years) and smallest in Southern Europe (difference of 0.1 years).

The trend in e0 after excluding lifestyle-attributable mortality was not only very similar for men and women across the 30 European countries (4.2 and 4.3 years, respectively); it was also more similar between countries and regions than all-cause mortality, at least for men (Figure 3). Among men, the variance between countries in e0 trends after excluding lifestyle-attributable mortality was 2.3, compared with 4.4 for all-cause mortality (Supplementary Table 2, available as Supplementary data at *IJE* online). Among women, the respective variances (2.0 vs 1.8) did not differ much. The increase in e0 was still far less favourable in Eastern Europe than in the other regions, even after excluding lifestyle-attributable mortality (Table 2). This seems mainly driven by the declines in e0 in the early 1990s (Figure 3). From around 2000, the increases in e0 were more similar between regions/countries.

## Discussion

The combined impact of smoking, obesity and alcohol on e0 declined among men from 6.6 years in 1990 to 5.8 years in 2014, mainly due to declining smoking-attributable mortality. Among women, the combined impact increased from 1.9 to 2.3 years due to mortality increases in all three lifestyle-related factors. The observed increase in e0 over the 1990–2014 period was 5.0 years for men and 4.0 years for women. Without the combined effect of these three factors, the increase in e0 would have been 4.2–4.3 years for both men and women, and would have been more similar between countries for men.

To estimate mortality attributable to smoking, obesity and alcohol combined, we multiplicatively aggregated the risk-factor-specific mortality fractions, assuming that the risk factors are independent and uncorrelated.^45^ In case the effects of these three lifestyle risk factors were to overlap, rather than having synergistic relations, their joint contribution would be overestimated. It is unknown how the above-mentioned bias is changing over time and consequently how this would affect our results on the combined impact of the three lifestyle factors on trends in life expectancy.

The techniques employed to estimate smoking-, obesity- and alcohol-attributable mortality were selected based on a careful assessment of different estimation techniques^48–50^ and data availability. However, these techniques provide estimates only and should be interpreted as such.

Comparing our estimates of the PGLE from the elimination of smoking-, obesity- and alcohol-attributable mortality with those using the Global Burden of Diseases 2017 data^44^ revealed that our estimates in 2014 for men were, on average, 0.3 years lower for obesity in Eastern Europe and for alcohol across all regions, but ∼0.7 years higher for smoking in CEE; for women, they were, on average, largely similar for alcohol, but 0.3–0.4 years lower for obesity in CEE and for smoking in Southern and Eastern Europe (see Supplementary Table 3, available as Supplementary data at *IJE* online). Based on this comparison, our estimates seem generally conservative.

The differences in the impact of lifestyle on trends in e0 between men (accelerating the increase) and women (decelerating the increase) are mainly attributable to large sex differences in the onset of the smoking epidemic. Because men generally started smoking in large numbers 20–30 years earlier than women did, trends in smoking-attributable mortality had been declining for several decades for men, but were still increasing for women in most countries.^15^^,^^16^ This earlier onset of smoking among men can be attributed to the earlier uptake of risky health behaviours by men than by women.^51^ However, women’s behaviours followed those of men when their roles in society changed due to women’s emancipation and rising female labour-force participation.^52^ The more recent trends in obesity- and alcohol-attributable mortality have been largely similar for men and women, in line with their relatively similar prevalence trends.^42^^,^^53^

The observed differences between regions and countries in the impact of lifestyle factors on trends in e0 can also be linked to international differences in the progression of the smoking epidemic. For example, the large impact of lifestyle factors on trends among Central European women can be attributed to the later start and the greater impact of the smoking epidemic.^16^ Large international differences in alcohol-prevalence trends, however, have also played a role. Among men, the high levels and increasing trends in alcohol prevalence until around 2005 in Eastern Europe^37^ led to a deceleration, instead of an acceleration, of life-expectancy increases. In contrast, among women in Southern Europe, declines in alcohol prevalence^54^ contributed to the relatively small negative impact of lifestyle on life-expectancy trends. These differences can be mainly attributed to differences in drinking cultures (spirits in Eastern Europe, wine in Southern Europe) and in economic development (economic hardship in Eastern Europe).^20^

We observed that the e0 trends for non-lifestyle-attributable mortality ran parallel for men and women, and were more similar between countries than the observed e0 trends for men. Moreover, from a longer-term perspective, the e0 trends after excluding lifestyle-attributable mortality seem relatively stable over time (Supplementary Figure 3, available as Supplementary data at *IJE* online). This generally uniform trend may capture the underlying gradual long-term increase in life expectancy in Europe, which, as postulated in the epidemiological transition theory,^55^ reflects the long-term effects of socio-economic growth and medical progress. In European countries, large increases in gross domestic product per capita since the late nineteenth century^56^ drove the secular-mortality decline during the twentieth century. From the 1950s onwards, increases in life expectancy were achieved primarily through declines in adult cardiovascular mortality linked to medical improvements (hypertension treatments, statins, thrombolysis, stents).^57^

Although country differences in e0 trends can be largely explained by the three lifestyle factors studied, important country differences in e0 for non-lifestyle-attributable mortality remain. Particularly notable are the less favourable trends in Eastern Europe (i.e. former Soviet republics). The declines in e0 in the early 1990s may be attributable to the economic and political disruptions that followed the dissolution of the Soviet Union.^21^^,^^58^^,^^59^ However, from 2000 onwards, the e0 trends without lifestyle-attributable mortality were again more similar between countries. This convergence might reflect increasing similarities between countries in factors such as healthcare seeking, health-related policies and socio-cultural trends.

Smoking, obesity and alcohol likely contribute not only to country differences in e0 trends, but also to socio-economic differences in e0 trends. The three lifestyle factors contribute substantially to socio-economic differences in mortality^60^ due to higher current prevalence and associated mortality among people with low than with high SES.^61^ This contribution has increased over time, due to socio-economic differences in the uptake of new behaviours, and those with a higher socio-economic position generally being the first to change to healthier behaviours again.^15^^,^^62^^,^^63^

Because lifestyle factors have a large effect on life-expectancy trends, it is important to consider them when studying the stagnation in the increase in life expectancy. In Europe—particularly in the UK, but also in France, Germany, Sweden and the Netherlands—slowdowns in life-expectancy increases have been observed since 2011.^24^^,^^25^ Increases in both obesity prevalence^25^ and alcohol abuse among UK adults^24^ have been mentioned as potential causes. For women in France, Germany, Sweden and the Netherlands, rapid increases in smoking-attributable mortality (Supplementary Figure 1b, available as Supplementary data at *IJE* online)^16^ are likely contributors as well. Decomposing recent mortality trends into lifestyle-attributable mortality and remaining mortality may help to explain recent e0 trends.

Similarly, when predicting future trends in life expectancy, the time-varying impact of lifestyle factors should be considered. Because mortality forecasts mostly rely on the extrapolation of past age-specific mortality trends,^64^ a major challenge is to identify an underlying mortality trend that is sufficiently stable to serve as the basis for extrapolation.^65^^,^^66^ At the same time, such extrapolation should not ignore the factors that may cause deviations from this underlying trend.^41^^,^^65^^,^^66^ Our results indicate, first, that the past trends in non-lifestyle-attributable mortality serve as a better baseline for extrapolations than all-cause mortality; and, second, that smoking, obesity and alcohol are factors that seem to cause important deviations that should be predicted separately using more advanced techniques. Indeed, over the short term, less favourable e0 trends are expected because continued increases in smoking-attributable mortality for women in most countries and general increases in obesity-attributable mortality are anticipated. Increases in e0 are expected to become more favourable again once the declines in smoking-attributable mortality among women become more widespread and the hypothesized decline in obesity-attributable mortality^17^^,^^18^^,^^28^ eventually sets in.

## Supplementary data

Supplementary data are available at *IJE* online.

## Author contributions

F.J. conceived of the study. F.J. designed the study with input from S.T.L. and A.E.K. F.J. ran the first analyses. S.T.L. ran the final analyses. All authors interpreted the results. F.J. drafted the manuscript. A.E.K. provided critical input on the draft manuscript. F.J. revised the manuscript. All authors reviewed and approved the final version of the manuscript.

## Funding

This work was supported by the Netherlands Organisation for Scientific Research (NWO) in relation to the research programme ‘Smoking, alcohol, and obesity, ingredients for improved and robust mortality projections’, under grant no. 452–13-001. See www.futuremortality.com.

## Supplementary Material

dyaa273_Supplementary_Data
