# Supplementary material for: The combined impact of smoking, obesity and alcohol on life-expectancy trends in Europe
Source: Int J Epidemiol. 2021 Jan 11;50(3):931–41. doi: 10.1093/ije/dyaa273 (PMC8271206; doi:10.1093/ije/dyaa273)
Supplement: dyaa273_Supplementary_Data [file dyaa273_supplementary_data.zip › ije-2020-05-0940-File002.pdf]

Supplementary data & methods

Setting

We estimated the combined impact of smoking, obesity, and alcohol abuse on trends in life expectancy at birth (e0) for the national populations by sex in 30 European countries, over the period 1990 up to 2014. For the selected countries, high-quality mortality and population data are available from the Human Mortality Database over a long period of time. We studied the impact not only by country, but also for the 30 countries combined, and for the five main European regions: Northern Europe (Denmark, Finland, Iceland, Norway, Sweden), Western Europe (Austria, Belgium, France, Germany, Ireland, Luxembourg, Netherlands, Switzerland, United Kingdom), Southern Europe (Greece, Italy, Portugal, Spain), Central Europe (Czech Republic, Hungary, Poland, Slovakia, Slovenia), and Eastern Europe (Belarus, Bulgaria, Estonia, Latvia, Lithuania, Ukraine, Russia).

In choosing our study period, we had to take into account the data availability of many different sources: for smoking-attributable mortality, for obesity-attributable mortality, for alcohol-attributable mortality, and for all-cause mortality. Moreover, as we wanted to perform our analysis not just for individual countries but as well for a vast majority of European countries combined, and for each region, we had to choose a period for which the abovementioned data was available in all the countries. We ended up with a study period from 1990 to 2014, because alcohol-attributable mortality could not be estimated before 1990, and smoking-attributable mortality could not be estimated after 2014 for several countries due to unavailability of lung cancer mortality rates from the WHO Mortality Database for more recent years. See Appendix S1 at the end of this document for the data availability by country for the different elements.

We present our results for life expectancy at birth instead of life expectancy at a later age, because life expectancy at birth is the most commonly used life expectancy measure.

Data

We used all-cause mortality and exposure data from the Human Mortality Database (downloaded 27 August 2018) by age (0-100+), sex, country, and year to estimate life expectancy at birth; to obtain all-age estimates of lifestyle-attributable mortality; and to obtain weighted averages across the 30 European countries, and across the countries within the five different European regions.

For the estimation of smoking-, obesity-, and alcohol-attributable mortality (see below), we used (i) lung cancer mortality data from the WHO Mortality Database (WHO Mortality Database update 11 April 2018), and additional epidemiological information on smoking from the American Cancer Society's Cancer Prevention Study II (ACS-CPSII) (Peto et al. 1992; Thun et al. 1997); (ii) alcohol-attributable mortality data from the Global Burden of Disease Study 2017 (ages 20+) (GBD 2017 Risk Factor Collaborators 2018; GBD 2019), adjusted for ages 65+ with the age pattern for alcohol-related causes of death (Semyonova et al. 2014) from the WHO Mortality Database (WHO Mortality Database 2018; accessed 11 April 2018) and the Human Cause of Death Database (Human Cause of Death Database, 2017); and (iii) obesity prevalence data from the NCD Risk Factor Collaboration study (NCD Risk Factor Collaboration 2017) and RRs of dying from obesity (DYNAMO-HIA Consortium 2010) to estimate obesity-attributable mortality. These data were available by sex and five-year age groups. We

obtained data from 1950 onwards for smoking, from 1975 onwards for obesity, and from 1990 onwards for alcohol.

Estimation smoking-, obesity-, and alcohol-attributable mortality

Deaths due to smoking, alcohol, and obesity at the population level cannot be directly measured, and need to be estimated. For each lifestyle factor (smoking, obesity, alcohol) a different estimation technique was selected based on a careful assessment of the pros and cons of the different approaches (Stoeldraijer et al. 2015, Trias-Llimos et al. 2018, Vidra et al. 2018), and on data availability. For estimating smoking-attributable mortality, a commonly applied indirect approach was selected that estimates exposure to smoking based on lung cancer mortality data adjusted for the part not due to smoking (Peto et al. 1992), and that subsequently applies all-cause mortality RRs (Janssen et al. 2013; Janssen 2020). For estimating obesity-attributable mortality, the population-attributable fraction formula (Rockhill et al. 1998) was applied directly to (estimated) prevalence data (NCD Risk Factor Collaboration 2017), thereby using all-cause RRs of dying from obesity (Vidra et al. 2019). For estimating alcohol-attributable mortality, data from the Global Burden of Disease 2017 study (GBD 2017 Risk Factor Collaborators 2018; GBD 2019) were used that were adjusted for higher ages using the age pattern (but not the level) observed for the main group of causes of death wholly attributable to alcohol (Janssen et al. 2020). For more details on each lifestyle factor, see below.

- *Smoking-attributable mortality*

We estimated smoking-attributable mortality fractions (SAMF) by country, year, sex, and age by applying our adapted and simplified indirect Peto-Lopez method (Peto et al. 1992; Janssen et al. 2013; Janssen 2020) to observed lung cancer mortality rates. This method takes into account that not all lung cancer deaths are attributable to smoking, and includes deaths from other causes that could be attributed to smoking.

First, the lifetime smoking prevalence by five-year age groups was estimated by comparing the observed national age- and sex-specific lung cancer mortality rates with the age- and sex-specific lung cancer rates of smokers and never-smokers (smoothed) from the ACS CPS-II study (Peto et al. 1992). We obtained the lifetime smoking prevalence by single year of age by means of Loess smoothing (span = 0.75; degree = 2), while using 85 as the age centre for the age group 80+. From age 86 onwards, we applied the smoothed lifetime smoking prevalence for age 85.

Second, we estimated the SAMF by single year of age (x) and sex (s) using the population attributable fraction (PAF) formula: $SAMF_{x,s} = p_{x,s} (RR_{x,s}-1)/(p_{x,s} (RR_{x,s}-1)+1)$, where $p_{x,s}$ reflects the lifetime smoking prevalence by single year of age and sex, and $RR_{x,s}$ reflects the relative risks of dying from smoking by single year of age and sex. RRs by five-year age groups (35-39, 40-44, …, 80-84, 85+) and sex were obtained by dividing the all-cause mortality rates among CPS-II current smokers by the all-cause mortality rates among CPS-II never smokers (Thun et al. 1997). To control for confounding – i.e., the exposure of smokers to other risk factors – we reduced the excess risk by 30%, in line with the largest reduction in excess risk that was observed for specific causes of death after controlling for confounding factors in the reanalysis of CPS-II by Thun et al. (2000) (Ezzati & Lopez 2003). By applying a second-degree polynomial, we obtained the RRs by single year of age, while using 86 as the age centre for 85+, and smoothing up until age 89 for men and age 86 for women, after which we kept the RRs stable.

Because smoking-attributable mortality before age 35 is very close to non-existent, smoking-attributable mortality is generally set to zero before age 35 (Peto et al. 1992), which we also did.

- *Alcohol-attributable mortality*

We used estimated alcohol-attributable mortality rates by five-year age groups (20-24,…, 90-94, 95+) from the Global Burden of Disease (GBD) study (GBD 2017 Risk Factor Collaborators 2018; GBD 2019), which were available from 1990 up until 2016. The GBD estimates comprise alcohol-related deaths from causes of death that are either wholly or partly related to alcohol, and the estimates are controlled for confounding (GBD 2017 Risk Factor Collaborators, 2018).

Because of quality issues with the GBD estimates for the highest ages (Trias-Llimós et al. 2018; Manthey & Rehm 2019), we applied to ages 65+ the age pattern observed for the main group of causes of death wholly attributable to alcohol (ICD-10 codes: F10, K70, X45, G312, G621, G721, I426, K292, K860, Q860, X65, and Y15) (Semyonova et al. 2014), mainly from the WHO Mortality Database (WHO Mortality Database 2018; accessed 11 April 2018); and, for Belarus, Russia, and Ukraine, from the Human Cause of Death Database (2017). More specifically, we calculated ratios for each country and sex, but for all years combined, between the five-year specific WHO alcohol-attributable mortality rates for ages 65-69 and above and the respective rates at ages 60-64, which we subsequently multiplied by the GBD alcohol-attributable mortality rate at ages 60-64 in order to obtain our final estimates for ages 65 and above.

We divided the resulting alcohol-attributable mortality rates by all-cause mortality rates from the Human Mortality Database (HMD, accessed 27 Sept 2018), and subsequently applied Loess smoothing to obtain alcohol-attributable mortality fractions by single year of age (ages 20-100).

- *Obesity-attributable mortality*

We used obesity prevalence data (BMI≥30kg/m2) by country, sex, age (20-24, …, 85+), and year (1975-2016) from the NCD Risk Factor Collaboration study (NCD Risk Factor Collaboration 2017). These data comprise the available measured height and weight data, supplemented with estimates from a Bayesian hierarchical model based on information from other years and related countries. We converted the obesity prevalence data by five-year age groups into single-age prevalence (20-100) by applying Loess smoothing, thereby using age 87.5 as the central age for 85+.

To obtain age- and sex-specific obesity-attributable mortality fractions ($OAMF_{x,s}$), we applied the population-attributable fraction formula (Rockhill et al. 1998) to the prevalence (p) data by single years of age: $OAMF_{x,s} = p_{x,s} (RR_{x,s}-1)/(p_{x,s} (RR_{x,s}-1)+1)$, where $RR_{x,s}$ are the relative risks of dying from obesity by single year of age and sex. These RRs were obtained by smoothing – using linear regression – the RRs by age group (<50, 50-59, 60-69, and ≥70 years) and sex from a review of studies mainly conducted in Western Europe and the USA (DYNAMO-HIA Consortium 2010). Their review included both studies that controlled for (different) confounding factors, such as smoking, and studies that did not. The overall RR of around 1.5 was largely in line with the overall European RR of 1.64 estimated by the Global BMI Mortality Collaboration (2016).

Estimation of combined lifestyle-attributable mortality

Based on these data, we calculated the age- and sex-specific lifestyle-attributable mortality fractions for smoking, obesity, and alcohol combined. Because alcohol-attributable mortality fractions were only available from 1990 onwards and smoking-attributable mortality could not be estimated after 2014 for several countries (see “setting” section above) we could estimate the combined lifestyle-

attributable mortality fractions for the period 1990 to 2014. In doing so, we set the obesity- and alcohol-attributable mortality fractions to zero for ages 0-19. Thus, strictly speaking we calculated the impact on life expectancy levels and trends of lifestyle-attributable mortality at adult ages (20+ for obesity, alcohol, and combined; 35+ for smoking), even as smoking, alcohol and obesity do not contribute substantially to mortality at lower ages.

To estimate the share of mortality due to smoking, alcohol, and obesity combined, we used the multiplicative aggregation of the fractions for the individual risk factors (Ezzati et al. 2003), using the following formula:

$$LAMF = PAF_{1..n} = 1 - \prod_{i=1}^{n}(1 - PAF_i)$$

where *i* stands for the individual risk factor, and $PAF_{1..n}$ stands for the lifestyle-attributable mortality fraction for the three lifestyle factors combined. We will refer to the latter as the lifestyle-attributable mortality fraction (LAMF) in the remainder of the text.

Although this aggregation rests on the strong assumption that risk factors are independent and uncorrelated (Ezzati et al. 2003), validation studies have demonstrated that estimates using this aggregation may closely resemble the true combined effect (Lim et al. 2015; GBD 2017 Risk Factor Collaborators 2018). Additional own analysis also revealed a strong congruency between the GBD 2017 PAF estimates for their nine behavioural risk factors, which were based on a more advanced method; and applying the multiplicative aggregation to the PAF for the nine behavioural risks separately (GBD 2017; see Appendix S2 at the end of this document). In case that the effects of these three lifestyle risk factors were to overlap, rather than having synergistic relations, their joint contribution would be overestimated.

Age-specific lifestyle-attributable mortality fractions and rates, by country and region

We ended up with age- and sex-specific smoking-, obesity-, alcohol-, and lifestyle-attributable mortality fractions up to age 100 (by single year of age) for each individual country. However, for our analysis, we also needed country-specific age- and sex-specific non-smoking-, non-obesity-, non-alcohol-, and non-lifestyle-attributable mortality rates as input for the life table calculations (see below). These were obtained by multiplying the respective HMD all-cause mortality rates (HMD update 27 August 2018) by one minus the different fractions.
In addition, we wanted to obtain estimates for all the 30 European countries combined, and for different European regions. We obtained weighted averages by first calculating the death numbers attributable to each lifestyle factor, and to the lifestyle factors combined. These figures were obtained by multiplying the smoking-, obesity-, alcohol-, and lifestyle-attributable mortality fractions by the respective HMD all-cause death numbers by single year of age (HMD update 27 August 2018).
By summing the attributable death numbers over the individual countries for the different regions, and subsequently dividing them by the aggregated all-cause death numbers and exposures, we obtained weighted averages of attributable mortality fractions and attributable mortality rates for the different regions, and for all countries combined.

Trends over time in lifestyle-attributable mortality fractions

To examine the trends over time in the smoking-, obesity-, alcohol-, and lifestyle-attributable mortality fractions, we obtained estimates across all ages (20-100) by applying direct standardisation using the age composition of deaths for the specific populations in 2010. We chose a population-specific standard over the application of one standard population (i.e. the average over the 30 European countries in 2010) because the results of the former proved more closely in line with the results for the potential gain in life expectancy. That is, a standard 'death population' can strongly influence country-specific results, because country differences in the composition of deaths can be large.

Potential gains in life expectancy (PGLE)

The contributions of the (combined) lifestyle factors to life expectancy levels in each of the European countries were assessed by means of the potential gains in life expectancy (PGLE)(Tsai et al. 1978) if smoking-, alcohol-, obesity-, and lifestyle-attributable mortality was eliminated. The PGLE were calculated by comparing the life expectancy at birth (e0) value for all-cause mortality (0-100+), with the e0 value based on life table calculations applied to non-smoking-, non-obesity-, non-alcohol-, and non-lifestyle-attributable mortality rates (0-100+). For the latter life table calculations, so-called associated single decrement life tables were constructed (Preston et al. 2001).

Effect on life expectancy trends

For our main aim of estimating the (combined) impact of smoking, obesity and alcohol on life expectancy trends in Europe, we graphically compared the trends over time in observed e0 (= for all-cause mortality) with the trends in estimated e0 values (= for non-smoking-, non-obesity-, non-alcohol-, and non-lifestyle-attributable mortality)(see before). To compare the overall change in observed e0 over the 1990-2014 period with this change in estimated e0 values, we subtracted the respective e0 values in 1990 from the respective e0 values in 2014.

Sensitivity analysis

We performed a sensitivity analysis in which we used the GBD estimates of smoking-, obesity-, and alcohol-attributable mortality instead of our estimates. More specifically, we compared for 2014 our estimates of the potential gain in life expectancy (PGLE) from the elimination of smoking-, obesity- and alcohol-attributable mortality using our estimates of smoking- obesity-, and alcohol-attributable mortality with similar estimates of PGLE based on the Global Burden of Disease 2017 estimates of smoking-, obesity-, and alcohol-attributable mortality (GBD 2018). For obesity-attributable mortality, the GBD estimates comprise estimates of the effect of high BMI (BMI of 22.5 kg/m^2 or greater). See Supplementary Table 3 for the outcome of our sensitivity analysis. Compared to the PGLE estimates using the GBD data on lifestyle-attributable mortality, our PGLE estimates in 2014 for men were, on average, 0.3 years lower for obesity in Eastern Europe and for alcohol across all regions, but approximately 0.7 years higher for smoking in CEE. For women, our PGLE estimates in 2014 were, on average, largely similar for alcohol, but 0.3-0.4 years lower for obesity in CEE and for smoking in Southern and Eastern Europe.

References

DYNAMO-HIA Consortium. Workpackage 7: Overweight and obesity. Report on data collection for overweight and obesity prevalence and related relative risks. 2010; Available at: www.dynamo-hia.eu.

Ezzati M, Hoorn SV, Rodgers A, Lopez AD, Mathers CD, Murray CJ, et al. Estimates of global and regional potential health gains from reducing multiple major risk factors. Lancet. 2003;362(9380):271-280.

Ezzati M, Lopez AD. Estimates of global mortality attributable to smoking in 2000. Lancet. 2003;362(9387):847-852.

GBD 2017 Risk Factor Collaborators. Global, regional, and national comparative risk assessment of 84 behavioural, environmental and occupational, and metabolic risks or clusters of risks for 195 countries and territories, 1990–2017: a systematic analysis for the Global Burden of Disease Study 2017. Lancet. 2018;392(10159):1923-1994.

Global BMI Mortality Collaborators. Body-mass index and all-cause mortality: individual-participant-data meta-analysis of 239 prospective studies in four continents. Lancet. 2016;388(10046):776-786.

Global Burden of Disease Study 2017. Results. Seattle, WA: Institute for Health Metrics and Evaluation (IHME), 2018. Available online https://gbd2017.healthdata.org/gbd-search/ (data downloaded on April 6, 2018).

Human Cause-of-Death Database. French Institute for Demographic Studies (France) and Max Planck Institute for Demographic Research (Germany). Available at www.causeofdeath.org (data downloaded on June 30, 2017).

Human Mortality Database. University of California, Berkeley (USA), and Max Planck Institute for Demographic Research (Germany). Available at: www.mortality.org (data downloaded on August 27, 2018).

Human Mortality Database. University of California, Berkeley (USA), and Max Planck Institute for Demographic Research (Germany). Available at: www.mortality.org (data downloaded on September 27, 2018).

Janssen F, El Gewily S, Bardoutsos A, Trias-Llimós S. Past and future alcohol-attributable mortality in Europe. Int J Environ Res Public Health. 2020;17(23);9024. doi: 10.3390/ijerph17239024.

Janssen F, van Wissen LJ, Kunst AE. Including the smoking epidemic in internationally coherent mortality projections. Demography. 2013;50:1341-62.

Janssen F. Similarities and differences between sexes and countries in the mortality imprint of the smoking epidemic in 34 low-mortality countries, 1950-2014. Nicotine Tob Res. 2020;22(7):1210-1220.

Lim SS, Carnahan E, Nelson EC, Gillespie CW, Mokdad AH, Murray CJ, et al. Validation of a new predictive risk model: measuring the impact of the major modifiable risks of death for patients and populations. Popul Health Metr. 2015;13;27. doi: 10.1186/s12963-015-0059-8.

Manthey J, Rehm J. Mortality from Alcoholic Cardiomyopathy: Exploring the Gap between Estimated and Civil Registry Data. J Clin Med. 2019;8(8);1137. doi:10.3390/jcm8081137.

NCD Risk Factor Collaboration (NCD-RisC). Worldwide trends in body-mass index, underweight, overweight, and obesity from 1975 to 2016: a pooled analysis of 2416 population-based measurement studies in 128.9 million children, adolescents, and adults. Lancet. 2017;390(10113):2627-2642.

Peto R, Boreham J, Lopez AD, et al. Mortality from tobacco in developed countries: indirect estimation from national vital statistics. Lancet. 1992;339:1268-78.

Preston SH, Heuveline P, Guillot M. Demography: Measuring and Modelling Population Processes. Oxford: Blackwell; 2000.

Rockhill B, Newman B, Weinberg C. Use and misuse of population attributable fractions. Am J Public Health. 1998;88:15–19.

Semyonova, V.G., Gavrilova, N.S., Sabgayda, T.P., Antonova, O.M., Nikitina, S.Y. & Evdokushkina, G.N. Approaches to the Assessment of Alcohol-Related Losses in the Russian Population. In: Anson, J. & Luy, M., editor. Mortality in an International Perspective. 1st ed. Dordrecht Heidelberg New York London: Springer; 2014. p. 137-168.

Stoeldraijer L, Bonneux L, van Duin C, van Wissen L, Janssen F. The future of smoking-attributable mortality: the case of England & Wales, Denmark and the Netherlands. Addiction. 2015;110(2):336-345.

Tsai SP, Lee ES, Hardy RJ. The effects of a reduction in leading causes of death: potential gains in life expectancy. Am J Public Health. 1978; 68: 966-71.Thun MJ, Apicella LF, Henley SJ. Smoking vs other risk factors as the cause of smoking-attributable mortality: confounding in the courtroom. JAMA. 2000; 284: 706–12.

Thun MJ, Day-Lally C, Myers DG, et al. Trends in tobacco smoking and mortality from cigarette use in Cancer Prevention Studies I (1959 through 1965) and II (1982 through 1988). In: Burns DM, Garfinkel L, Samet JM, eds. Changes in Cigarette-Related Disease Risks and Their Implications for Prevention and Control. Bethesda, Md: National Cancer Institute: Smoking and Tobacco Control Monograph. 1997:305-82.

Trias-Llimós S, Martikainen P, Mäkelä P, Janssen F. Comparison of different approaches for estimating age-specific alcohol-attributable mortality: The cases of France and Finland. PLoS One. 2018;13(3):e0194478. doi: 10.1371/journal.pone.0194478.

Vidra N, Bijlsma MJ, Janssen F. Impact of Different Estimation Methods on Obesity-Attributable Mortality Levels and Trends: The Case of The Netherlands. Int J Environ Res Public Health. 2018 Sep 29;15(10):2146. doi: 10.3390/ijerph15102146.

Vidra N, Trias-Llimos S, Janssen F. Impact of obesity on life expectancy among different European countries: secondary analysis of population-level data over the 1975-2012 period. BMJ Open. 2019 Jul 31;9(7):e028086. doi: 10.1136/bmjopen-2018-028086.

World Health Organization. WHO Mortality Database. Available at: http://www.who.int/healthinfo/statistics/mortality_rawdata/en/ (update April 11, 2018).

Appendix S1 – Availability of smoking-, obesity- and alcohol-attributable mortality estimates and all-cause mortality, by country

Country	Region	Smoking-attributable mortality		Obesity-attributable mortality		Alcohol-attributable mortality		All-cause mortality	
		Start year	End year	Start year	End year	Start year	End year	Start year	End year
Austria	West	1955	2014	1975	2016	1990	2017	1947	2017
Belarus	East	1981	2014	1975	2016	1990	2016	1959	2016
Belgium	West	1954	2015	1975	2016	1990	2015	1841	2015
Bulgaria	East	1964	2010	1975	2016	1990	2010	1947	2010
Czech Republic	Central	1953	2016	1975	2016	1990	2016	1950	2016
Denmark	North	1951	2015	1975	2016	1990	2016	1835	2016
Estonia	East	1981	2014	1975	2016	1990	2017	1959	2017
Finland	North	1952	2015	1975	2016	1990	2015	1878	2015
France	West	1950	2015	1975	2016	1990	2016	1816	2016
Germany	West	1970	2015	1975	2016	1990	2017	1956	2017
Greece	South	1961	2013	1975	2016	1990	2013	1981	2013
Hungary	Central	1955	2014	1975	2016	1990	2017	1950	2017
Iceland	North	1951	2016	1975	2016	1990	2016	1838	2016
Ireland	West	1950	2014	1975	2016	1990	2014	1950	2014
Italy	South	1951	2014	1975	2016	1990	2014	1872	2014
Latvia	East	1980	2014	1975	2016	1990	2017	1959	2017
Lithuania	East	1981	2014	1975	2016	1990	2017	1959	2017
Luxembourg	West	1967	2014	1975	2016	1990	2014	1960	2014
Netherlands	West	1950	2016	1975	2016	1990	2016	1850	2016
Norway	North	1951	2014	1975	2016	1990	2014	1846	2014
Poland	Central	1959	2014	1975	2016	1990	2016	1958	2016
Portugal	South	1955	2015	1975	2016	1990	2015	1940	2015
Russia	East	1980	2013	1975	2016	1990	2014	1959	2014
Slovakia	Central	1953	2014	1975	2016	1990	2017	1950	2017
Slovenia	Central	1985	2014	1975	2016	1990	2017	1983	2017

Country	Region	Smoking-attributable mortality		Obesity-attributable mortality		Alcohol-attributable mortality		All-cause mortality	
		Start year	End year	Start year	End year	Start year	End year	Start year	End year
Spain	South	1951	2014	1975	2016	1990	2016	1908	2017
Sweden	North	1951	2016	1975	2016	1990	2017	1751	2017
Switzerland	West	1951	2015	1975	2016	1990	2016	1876	2016
Ukraine	East	1981	2012	1975	2016	1990	2013	1959	2013
United Kingdom	West	1950	2015	1975	2016	1990	2016	1922	2016

Appendix S2 - Comparison of the population-attributable fraction (PAF) estimate for nine behavioural risks factors combined directly from the GBD with the PAF estimate when applying the multiplicative aggregation to the PAF for the nine behavioural risks separately

For this additional analysis, we used the estimates from the Global Burden of Disease Study 2017 (GBD 2017), which are available online at http://ghdx.healthdata.org/gbd-results-tool.

We performed the comparison based on data for men in the Netherlands in 2010.

The population-attributable fractions for the three level 1 risk factors they distinguished were:

**Level 1 risk factors GBD**	**PAF GBD**
Environmental/Occupational risks	0.125
Behavioural risk	0.45
Metabolic risks	0.30

This behavioural risk estimate comprises the estimate for nine behavioural risk factors (see below) combined.

The population-attributable fractions for the level 2 risk factors within “behavioural risk” were:

**Behavioural risk factors**	**PAF GBD**
Dietary risks	0.15
Child and maternal malnutrition	0.00
Tobacco	0.28
Alcohol use	0.06
Drug use	0.01
Low physical activity	0.02
Unsafe sex	0.00
Childhood maltreatment	0.00
Intimate partner violence	0.00

Applying the multiplicative assumption to the abovementioned level 2 risk factor PAFs resulted in a combined PAF of 0.44, which was almost identical to the 0.45 reported by GBD.
