# Supplementary material for: The combined impact of smoking, obesity and alcohol on life-expectancy trends in Europe
Source: Int J Epidemiol. 2021 Jan 11;50(3):931–41. doi: 10.1093/ije/dyaa273 (PMC8271206; doi:10.1093/ije/dyaa273)

Supplementary Tables and Figures

Supplementary Table S1a - Impact of smoking, obesity, and alcohol (separately and combined) on life expectancy at birth (e0) for the 30 individual European countries, 1990 and 2014*, men. By means of estimates of the potential gain in life expectancy (PGLE) (in years) from the elimination of mortality linked to the respective lifestyle factor. Lifestyle refers to smoking, obesity and alcohol combined.

			PGLE 1990 (in years)					PGLE 2014* (in years)			
Region	Country	eo 1990	lifestyle	smoking	obesity	alcohol	eo 2014*	lifestyle	smoking	obesity	alcohol
North	Denmark	72.0	5.4	4.0	0.7	1.1	78.6	3.8	2.1	1.0	1.1
North	Finland	70.9	5.7	4.0	0.8	1.4	78.1	3.7	1.5	1.2	1.3
North	Iceland	75.4	3.0	2.2	0.7	0.2	81.1	2.5	1.3	1.0	0.4
North	Norway	73.4	3.2	2.4	0.6	0.4	80.0	2.8	1.4	1.0	0.5
North	Sweden	74.8	2.7	1.7	0.6	0.5	80.4	2.3	0.9	0.9	0.6
West	Austria	72.2	5.4	3.7	0.6	1.6	78.9	3.8	2.0	1.0	1.1
West	Belgium	72.7	6.0	4.8	0.8	0.8	78.6	4.3	2.6	1.2	1.0
West	France	72.7	6.0	4.0	0.7	2.1	79.3	4.4	2.6	1.1	1.3
West	Germany	71.9	5.6	3.8	0.8	1.5	78.4	4.1	2.2	1.2	1.1
West	Ireland	72.1	4.5	3.4	0.6	0.7	79.2	3.5	1.7	1.1	0.9
West	Luxembourg	72.3	6.1	4.5	0.7	1.3	79.1	4.0	2.3	1.1	1.1
West	Netherlands	73.8	5.2	4.4	0.5	0.5	79.9	3.3	2.1	0.9	0.6
West	Switzerland	74.0	4.4	3.2	0.6	0.9	80.9	2.7	1.4	0.9	0.6
West	United Kingdom	72.8	5.0	4.0	0.7	0.4	79.3	3.5	1.9	1.3	0.6
South	Greece	74.7	4.7	3.5	0.6	1.0	78.5	4.7	3.2	1.2	0.7
South	Italy	73.6	5.5	4.0	0.7	1.3	80.6	3.3	1.9	0.9	0.6
South	Portugal	70.8	4.6	2.3	0.5	2.3	77.9	4.2	2.3	1.0	1.5
South	Spain	73.4	5.3	3.5	0.8	1.7	80.1	4.2	2.4	1.2	1.0
Central	Czech Republic	67.5	7.6	5.8	1.0	1.5	75.7	4.9	2.7	1.4	1.3
Central	Hungary	65.1	9.3	6.4	1.1	2.9	72.3	7.8	5.3	1.7	1.9
Central	Poland	66.3	7.3	5.8	0.9	1.1	73.7	6.4	4.0	1.5	1.6
Central	Slovakia	66.5	8.0	5.9	0.8	2.1	73.3	5.6	3.2	1.2	1.7
Central	Slovenia	69.8	6.6	5.0	0.6	1.5	78.0	4.0	2.6	1.0	0.8
East	Belarus	66.2	7.5	5.4	0.8	2.1	67.8	7.6	4.2	1.5	2.8
East	Bulgaria	68.1	5.8	3.9	0.8	1.7	70.3	5.9	4.0	1.3	1.2
East	Estonia	64.7	7.2	5.8	0.9	0.9	72.3	6.6	3.6	1.3	2.3
East	Latvia	64.1	7.6	5.9	0.9	1.4	69.1	7.4	4.2	1.5	2.5
East	Lithuania	66.4	8.0	5.9	1.0	2.0	69.1	8.1	4.5	1.7	2.9
East	Russia	63.8	7.9	6.1	0.7	1.6	65.1	8.4	4.7	1.4	3.1
East	Ukraine	65.6	7.7	5.6	0.8	2.0	66.1	7.8	4.4	1.5	2.9

* Or latest available year before that: Bulgaria (2010), Greece (2013), Ukraine (2012) and Russia (2013)

Supplementary Table S1b - Impact of smoking, obesity, and alcohol (separately and combined) on life expectancy at birth (e0) for the 30 individual European countries, 1990 and 2014*, women. By means of estimates of the potential gain in life expectancy (PGLE) (in years) from the elimination of mortality linked to the respective lifestyle factor. Lifestyle refers to smoking, obesity and alcohol combined.

			PGLE 1990 (in years)					PGLE 2014* (in years)			
**Region**	**Country**	**eo 1990**	**lifestyle**	**smoking**	**obesity**	**alcohol**	**eo 2014***	**lifestyle**	**smoking**	**obesity**	**alcohol**
**North**	Denmark	77.7	3.2	2.3	0.6	0.5	82.7	3.3	2.4	0.7	0.4
**North**	Finland	78.9	1.4	0.4	0.7	0.2	83.9	1.9	0.8	0.9	0.3
**North**	Iceland	80.6	2.5	1.8	0.7	0.1	84.1	2.6	1.8	0.8	0.1
**North**	Norway	79.8	1.6	0.7	0.7	0.2	84.1	2.5	1.4	0.9	0.2
**North**	Sweden	80.4	1.4	0.6	0.6	0.3	84.1	2.1	1.2	0.7	0.3
**West**	Austria	78.9	1.5	0.6	0.5	0.4	83.7	2.0	1.0	0.7	0.3
**West**	Belgium	79.3	1.6	0.5	0.8	0.4	83.5	2.3	1.1	1.0	0.4
**West**	France	81.0	1.4	0.2	0.6	0.6	85.5	1.8	0.8	0.8	0.4
**West**	Germany	78.4	1.8	0.5	0.8	0.5	83.4	2.3	1.1	1.0	0.3
**West**	Ireland	77.7	2.6	1.8	0.6	0.3	83.2	3.0	1.9	1.0	0.3
**West**	Luxembourg	78.2	1.6	0.6	0.7	0.4	84.5	2.2	1.1	0.8	0.4
**West**	Netherlands	80.1	1.5	0.7	0.6	0.3	83.3	2.8	1.7	1.0	0.3
**West**	Switzerland	80.7	1.3	0.4	0.5	0.4	85.1	1.6	0.8	0.6	0.3
**West**	United Kingdom	78.5	2.9	2.1	0.8	0.2	83.0	3.1	1.9	1.0	0.3
**South**	Greece	79.7	1.4	0.4	0.8	0.2	83.7	1.6	0.6	1.0	0.1
**South**	Italy	80.3	1.6	0.4	0.7	0.4	85.2	1.6	0.7	0.8	0.2
**South**	Portugal	77.8	1.3	0.1	0.6	0.6	84.2	1.3	0.2	0.8	0.3
**South**	Spain	80.6	1.4	0.0	0.9	0.5	85.6	1.6	0.3	1.1	0.3
**Central**	Czech Republic	75.4	2.0	0.7	1.1	0.3	81.7	2.3	1.1	1.1	0.3
**Central**	Hungary	73.8	3.1	1.5	1.0	0.7	79.2	3.8	2.5	1.2	0.4
**Central**	Poland	75.3	1.9	0.8	1.1	0.1	81.4	2.8	1.5	1.2	0.3
**Central**	Slovakia	75.4	1.4	0.4	0.8	0.2	80.3	1.8	0.7	0.9	0.2
**Central**	Slovenia	77.7	1.6	0.5	0.7	0.4	83.7	2.0	1.1	0.8	0.2
**East**	Belarus	75.8	1.6	0.1	1.1	0.4	78.4	1.8	0.0	1.3	0.6
**East**	Bulgaria	74.8	1.4	0.2	0.9	0.3	77.3	1.4	0.3	1.0	0.1
**East**	Estonia	74.9	1.7	0.5	1.1	0.2	81.5	2.2	0.7	1.1	0.5
**East**	Latvia	74.5	1.8	0.5	1.2	0.2	79.2	1.9	0.3	1.2	0.5
**East**	Lithuania	76.2	1.9	0.3	1.3	0.3	79.9	2.1	0.4	1.4	0.5
**East**	Russia	74.3	2.1	0.5	1.4	0.3	76.3	2.4	0.2	1.6	0.7
**East**	Ukraine	74.9	2.0	0.5	1.1	0.5	76.0	2.2	0.2	1.3	0.8

* Or latest available year before that: Bulgaria (2010), Greece (2013), Ukraine (2012) and Russia (2013)

Supplementary Table S2 Impact of smoking, obesity and alcohol (separately and combined) on the change in life expectancy at birth (e0) in 30 European countries from 1990 until 2014*, by sex and country. Lifestyle refers to smoking, obesity and alcohol combined.

		Men					Women				
		Change in eo 1990-2014* (in years)					Change in eo 1990-2014* (in years)				
**Euro-pean region**	**Country**	Observed	Without lifestyle	Without smoking	Without obesity	Without alcohol	Observed	Without lifestyle	Without smoking	Without obesity	Without alcohol
**North**	Denmark	6.54	4.99	4.64	6.90	6.51	4.94	5.02	5.03	5.04	4.82
**North**	Finland	7.19	5.19	4.73	7.62	7.13	4.96	5.55	5.32	5.16	5.06
**North**	Iceland	5.78	5.31	4.86	6.08	5.98	3.52	3.65	3.55	3.59	3.56
**North**	Norway	6.58	6.10	5.63	7.00	6.66	4.29	5.21	4.99	4.53	4.33
**North**	Sweden	5.54	5.09	4.73	5.88	5.56	3.66	4.36	4.27	3.81	3.63
**West**	Austria	6.69	5.08	5.04	7.08	6.21	4.87	5.30	5.31	5.05	4.75
**West**	Belgium	5.88	4.22	3.66	6.28	6.05	4.19	4.90	4.84	4.32	4.21
**West**	France	6.56	5.00	5.24	6.98	5.71	4.48	4.87	5.07	4.62	4.21
**West**	Germany	6.52	5.01	4.90	6.97	6.06	4.93	5.47	5.50	5.14	4.75
**West**	Ireland	7.03	5.97	5.38	7.55	7.14	5.52	5.92	5.63	5.85	5.53
**West**	Luxembourg	6.83	4.80	4.57	7.24	6.56	6.25	6.92	6.78	6.37	6.32
**West**	Netherlands	6.05	4.20	3.69	6.53	6.11	3.20	4.50	4.28	3.56	3.16
**West**	Switzerland	6.98	5.27	5.19	7.32	6.62	4.40	4.77	4.83	4.55	4.22
**West**	United Kingdom	6.41	4.90	4.23	6.95	6.60	4.49	4.68	4.36	4.77	4.57
**South**	Greece	3.79	3.77	3.57	4.31	3.53	4.05	4.25	4.22	4.19	3.97
**South**	Italy	6.92	4.70	4.86	7.21	6.20	4.92	4.99	5.17	5.02	4.64
**South**	Portugal	7.16	6.81	7.13	7.69	6.43	6.35	6.37	6.50	6.58	6.00
**South**	Spain	6.65	5.47	5.60	7.04	6.00	5.06	5.23	5.37	5.19	4.84
**Central**	Czech Republic	8.19	5.51	5.13	8.54	7.99	6.33	6.62	6.68	6.31	6.34
**Central**	Hungary	7.12	5.64	6.00	7.67	6.12	5.47	6.19	6.51	5.61	5.13
**Central**	Poland	7.41	6.55	5.60	8.00	7.90	6.14	7.04	6.86	6.27	6.30
**Central**	Slovakia	6.74	4.32	4.03	7.18	6.28	4.96	5.37	5.28	5.07	4.99
**Central**	Slovenia	8.20	5.65	5.79	8.53	7.44	5.99	6.40	6.59	6.04	5.79
**East**	Belarus	1.57	1.64	0.34	2.30	2.24	2.64	2.90	2.52	2.82	2.86
**East**	Bulgaria	2.23	2.35	2.29	2.75	1.72	2.45	2.55	2.54	2.60	2.30
**East**	Estonia	7.60	7.03	5.37	8.06	9.00	6.60	7.00	6.80	6.55	6.90
**East**	Latvia	4.93	4.73	3.23	5.51	6.03	4.72	4.85	4.59	4.76	4.98
**East**	Lithuania	2.73	2.78	1.34	3.38	3.70	3.70	3.90	3.72	3.74	3.89
**East**	Russia	1.35	1.78	-0.11	2.01	2.82	1.97	2.29	1.72	2.14	2.42
**East**	Ukraine	0.47	0.61	-0.76	1.11	1.32	1.09	1.22	0.78	1.27	1.37
	**Variance**	4.36	2.30	3.48	4.04	3.39	1.81	1.99	2.31	1.76	1.66

* Or up to latest available year before 2014: Bulgaria (2010), Greece (2013), Ukraine (2012) and Russia (2013)

Supplementary Table S3 - Comparison of the impact of smoking, obesity and alcohol on life expectancy at birth* based on our own versus the Global Burden of Disease (GBD) estimates of smoking- obesity- and alcohol-attributable mortality, 2014 or latest available year, by country and sex. *By means of estimates of the potential gain in life expectancy (PGLE)(in years) from the elimination of mortality linked to the respective lifestyle factor.

a) Men

Region	Country	Year	Smoking own	Smoking GBD	Obesity own	Obesity GBD	Alcohol own	Alcohol GBD
**North**	Denmark	2014	2.12	2.57	1.01	0.75	1.06	1.29
**North**	Finland	2014	1.51	1.47	1.20	0.99	1.34	1.45
**North**	Iceland	2014	1.26	1.83	0.99	1.09	0.40	0.41
**North**	Norway	2014	1.40	1.98	1.04	0.75	0.47	0.50
**North**	Sweden	2014	0.92	1.56	0.94	0.86	0.56	0.64
**West**	Austria	2014	2.03	2.16	1.01	1.03	1.11	1.37
**West**	Belgium	2014	2.58	3.05	1.16	0.78	1.01	1.22
**West**	France	2014	2.63	2.47	1.13	0.78	1.26	1.61
**West**	Germany	2014	2.19	2.28	1.22	1.01	1.06	1.24
**West**	Ireland	2014	1.74	2.33	1.13	0.91	0.86	1.19
**West**	Luxembourg	2014	2.28	2.08	1.11	0.84	1.07	1.68
**West**	Netherlands	2014	2.08	2.54	0.93	0.70	0.55	0.69
**West**	Switzerland	2014	1.41	2.03	0.90	0.72	0.55	0.63
**West**	United Kingdom	2014	1.86	1.9	1.27	0.99	0.64	0.74
**South**	Greece	2013	3.23	3.94	1.17	1.28	0.75	1.04
**South**	Italy	2014	1.93	2.24	0.95	0.90	0.59	0.83
**South**	Portugal	2014	2.29	2.22	1.01	0.88	1.52	2.16
**South**	Spain	2014	2.40	3.01	1.22	0.95	1.01	1.47
**Central**	Czech Republic	2014	2.70	2.91	1.39	1.29	1.26	1.70
**Central**	Hungary	2014	5.29	3.62	1.66	1.60	1.93	2.47
**Central**	Poland	2014	3.99	3.37	1.49	1.43	1.64	1.95
**Central**	Slovakia	2014	3.24	2.95	1.24	1.45	1.68	1.95
**Central**	Slovenia	2014	2.62	2.32	0.97	1.06	0.76	0.83
**East**	Belarus	2014	4.18	4.24	1.49	1.78	2.80	3.43
**East**	Bulgaria	2010	3.98	3.88	1.35	1.92	1.22	1.47
**East**	Estonia	2014	3.57	2.86	1.31	1.67	2.32	2.88
**East**	Latvia	2014	4.18	3.3	1.45	1.65	2.49	2.81
**East**	Lithuania	2014	4.47	3.25	1.67	1.53	2.93	3.51
**East**	Russia	2013	4.69	4.18	1.42	1.68	3.06	3.42
**East**	Ukraine	2012	4.43	3.99	1.46	1.55	2.88	3.43
Europe		2010	3.76	3.39	1.28	1.33	2.08	2.41
Northern Europe		2014	1.40	1.83	1.03	0.85	0.83	0.94
Western Europe		2014	2.19	2.28	1.18	0.92	0.96	1.16
Southern Europe		2013	2.30	2.67	1.05	0.94	0.84	1.19
Central Europe		2014	3.88	3.27	1.47	1.43	1.61	1.98
Eastern Europe		2010	4.96	4.12	1.35	1.63	3.41	3.75

b) Women

Region	Country	Year	Smoking Own	Smoking GBD	Obesity Own	Obesity GBD	Alcohol Own	Alcohol GBD
**North**	Denmark	2014	2.36	2.23	0.74	0.66	0.41	0.50
**North**	Finland	2014	0.76	0.64	0.92	0.93	0.34	0.27
**North**	Iceland	2014	1.82	1.19	0.78	0.73	0.13	0.05
**North**	Norway	2014	1.44	1.41	0.95	0.69	0.21	0.12
**North**	Sweden	2014	1.23	1.46	0.72	0.81	0.28	0.33
**West**	Austria	2014	1.05	1.10	0.72	0.99	0.31	0.32
**West**	Belgium	2014	1.14	1.49	0.95	0.78	0.39	0.45
**West**	France	2014	0.75	0.98	0.77	0.80	0.36	0.42
**West**	Germany	2014	1.07	1.13	1.03	0.97	0.34	0.35
**West**	Ireland	2014	1.89	1.64	0.97	0.78	0.30	0.45
**West**	Luxembourg	2014	1.15	1.40	0.78	1.13	0.44	0.61
**West**	Netherlands	2014	1.75	1.74	0.98	0.70	0.26	0.27
**West**	Switzerland	2014	0.83	1.06	0.61	0.71	0.27	0.26
**West**	United Kingdom	2014	1.92	1.46	1.04	0.86	0.27	0.26
**South**	Greece	2013	0.56	1.25	0.96	1.11	0.13	0.05
**South**	Italy	2014	0.70	0.88	0.84	0.95	0.17	0.21
**South**	Portugal	2014	0.23	0.41	0.83	0.89	0.27	0.43
**South**	Spain	2014	0.31	0.70	1.05	1.00	0.26	0.38
**Central**	Czech Republic	2014	1.06	1.31	1.07	1.38	0.30	0.32
**Central**	Hungary	2014	2.50	1.88	1.19	1.58	0.39	0.44
**Central**	Poland	2014	1.49	1.50	1.20	1.49	0.30	0.24
**Central**	Slovakia	2014	0.74	0.91	0.92	1.64	0.22	0.03
**Central**	Slovenia	2014	1.13	1.02	0.78	1.12	0.21	0.16
**East**	Belarus	2014	0.01	0.76	1.25	1.80	0.63	0.66
**East**	Bulgaria	2010	0.33	1.54	1.03	1.81	0.13	0.06
**East**	Estonia	2014	0.71	0.79	1.07	1.88	0.46	0.55
**East**	Latvia	2014	0.35	0.74	1.19	1.77	0.46	0.33
**East**	Lithuania	2014	0.36	0.61	1.35	1.58	0.51	0.44
**East**	Russia	2013	0.25	0.58	1.55	1.88	0.74	0.73
**East**	Ukraine	2012	0.17	0.54	1.27	1.81	0.81	0.95
Europe		2010	0.71	0.95	1.18	1.37	0.52	0.53
Northern Europe		2014	1.40	1.42	0.81	0.78	0.31	0.32
Western Europe		2014	1.24	1.23	0.95	0.88	0.32	0.34
Southern Europe		2013	0.51	0.81	0.91	0.97	0.21	0.28
Central Europe		2014	1.53	1.48	1.14	1.49	0.31	0.27
Eastern Europe		2010	0.22	0.60	1.46	1.86	0.85	0.87

Supplementary Figure S1 - Trends over time in age-standardised smoking-, obesity-, and alcohol-attributable mortality fractions (separately and combined)(%), ages 20-100, by country, 1990-2014*. * Or latest available year before that: Bulgaria (2010), Greece (2013), Ukraine (2012) and Russia (2013). Lifestyle refers to smoking, obesity and alcohol combined.

a) MEN

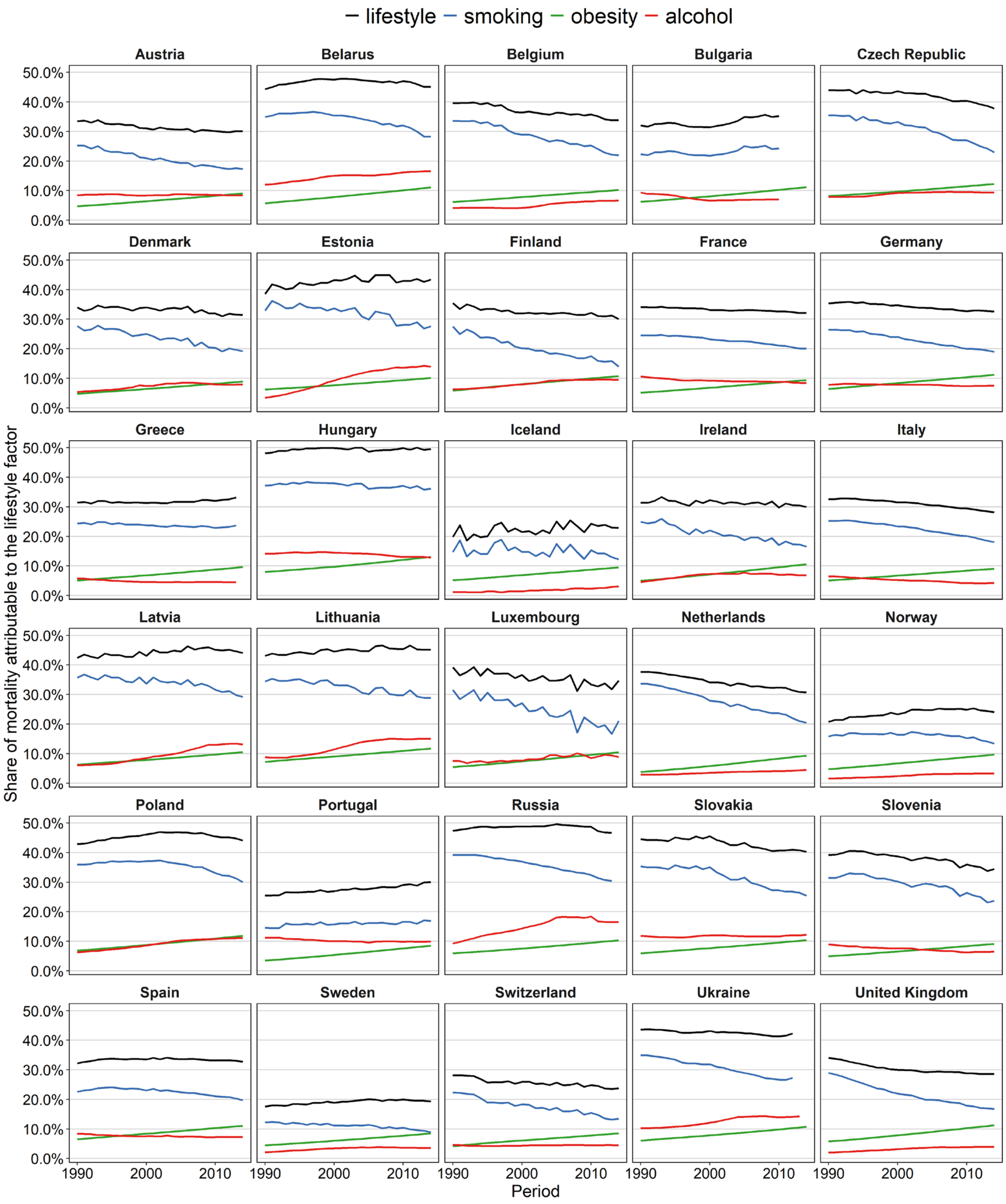

b) WOMEN

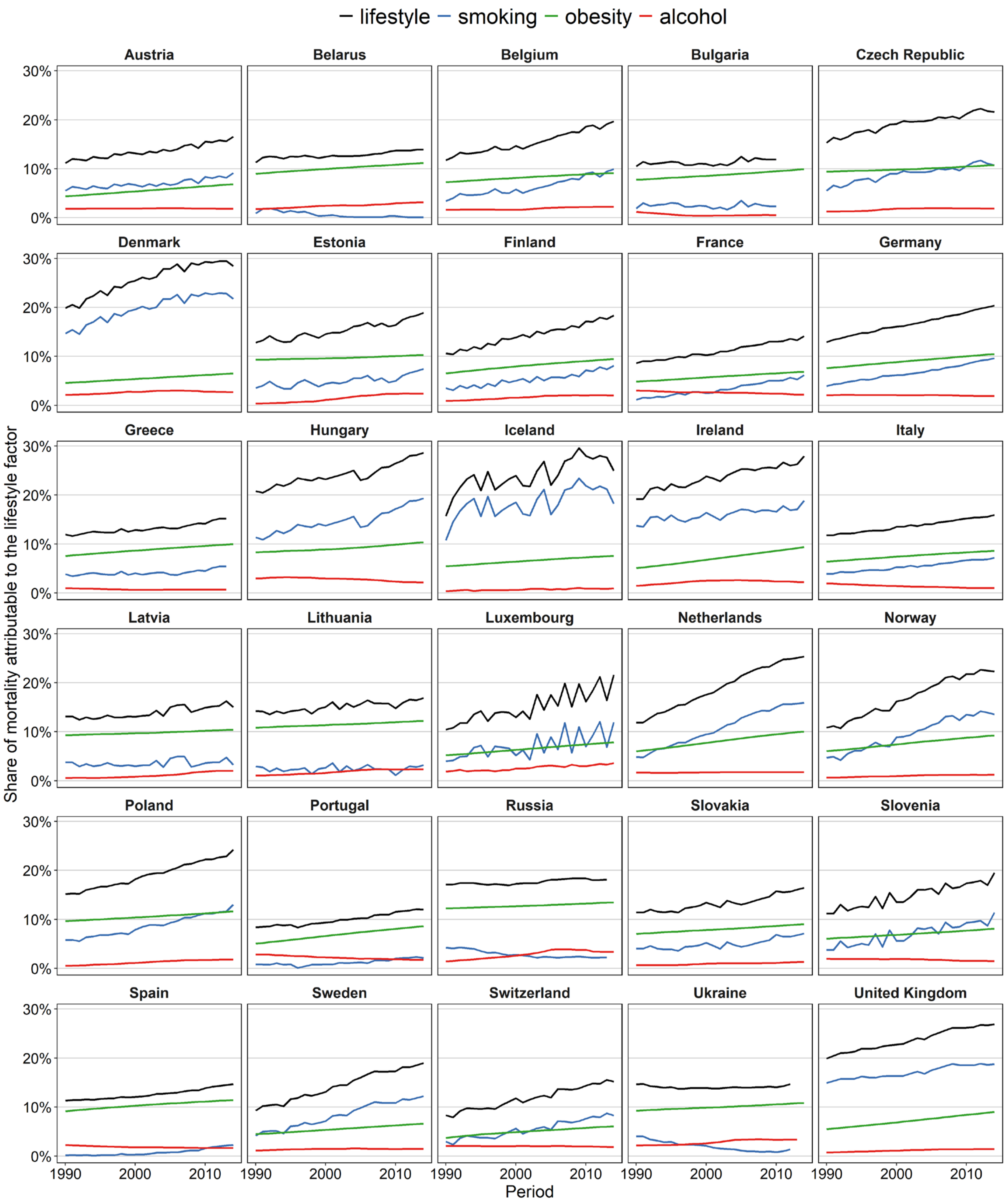
lifestyle smoking obesity alcohol
Austria
Belarus
Belgium
Bulgaria
Czech Republic
Denmark
Estonia
Finland
France
Germany
Greece
Hungary
Iceland
Ireland
Italy
Latvia
Lithuania
Luxembourg
Netherlands
Norway
Poland
Portugal
Russia
Slovakia
Slovenia
Spain
Sweden
Switzerland
Ukraine
United Kingdom
0%
10%
20%
30%
1990
2000
2010
Share of mortality attributable to the lifestyle factor
Period

Supplementary Figure S2 - Trends over time in the potential gain in life expectancy (PGLE) when eliminating smoking-, obesity- and alcohol-attributable mortality (separately and combined), 1990-2014*, by country and sex. * Or latest available year before that: Bulgaria (2010), Greece (2013), Ukraine (2012) and Russia (2013). Lifestyle-attributable mortality refers to mortality that is attributable to smoking, obesity and alcohol combined.

a) MEN

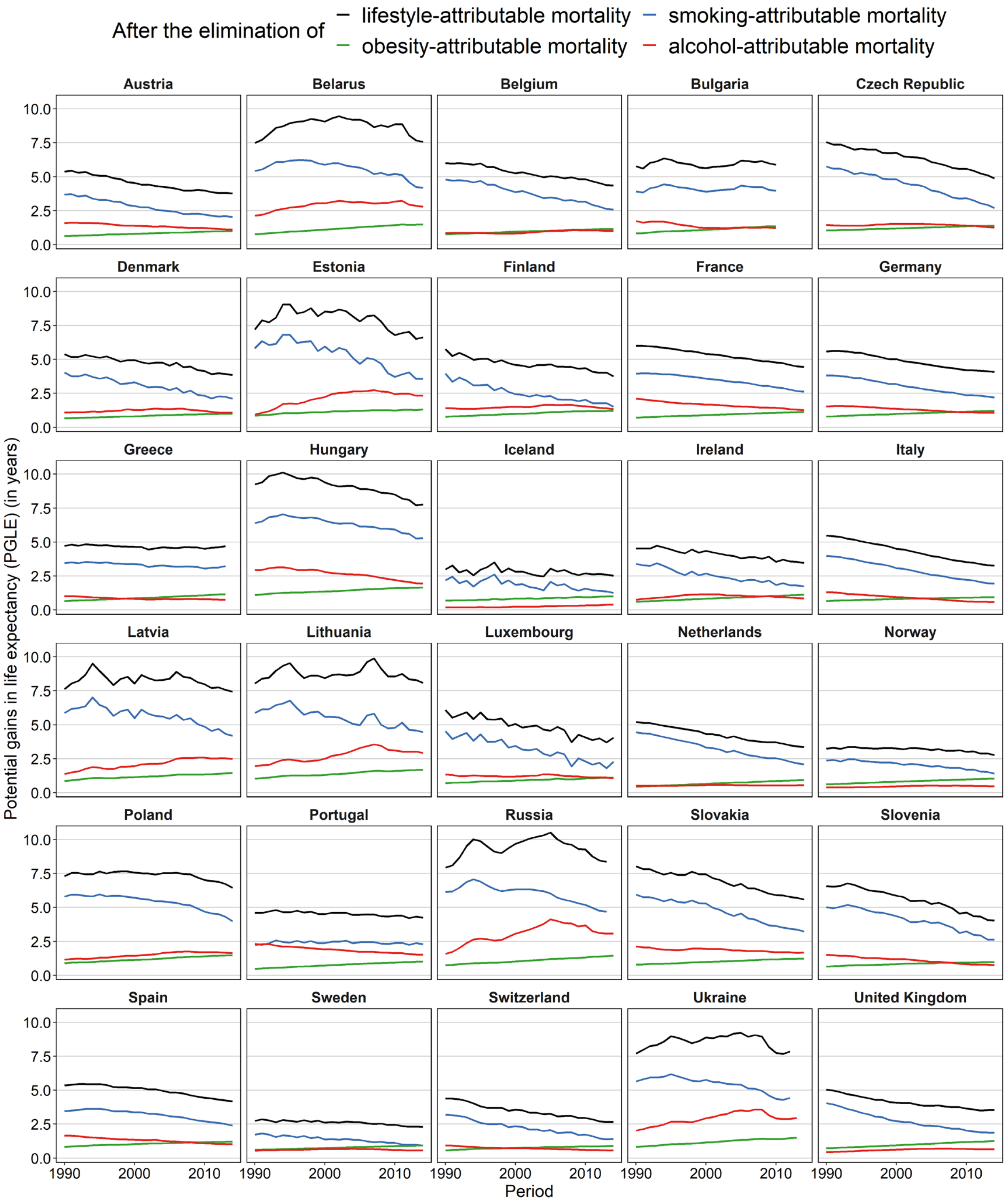

b) WOMEN

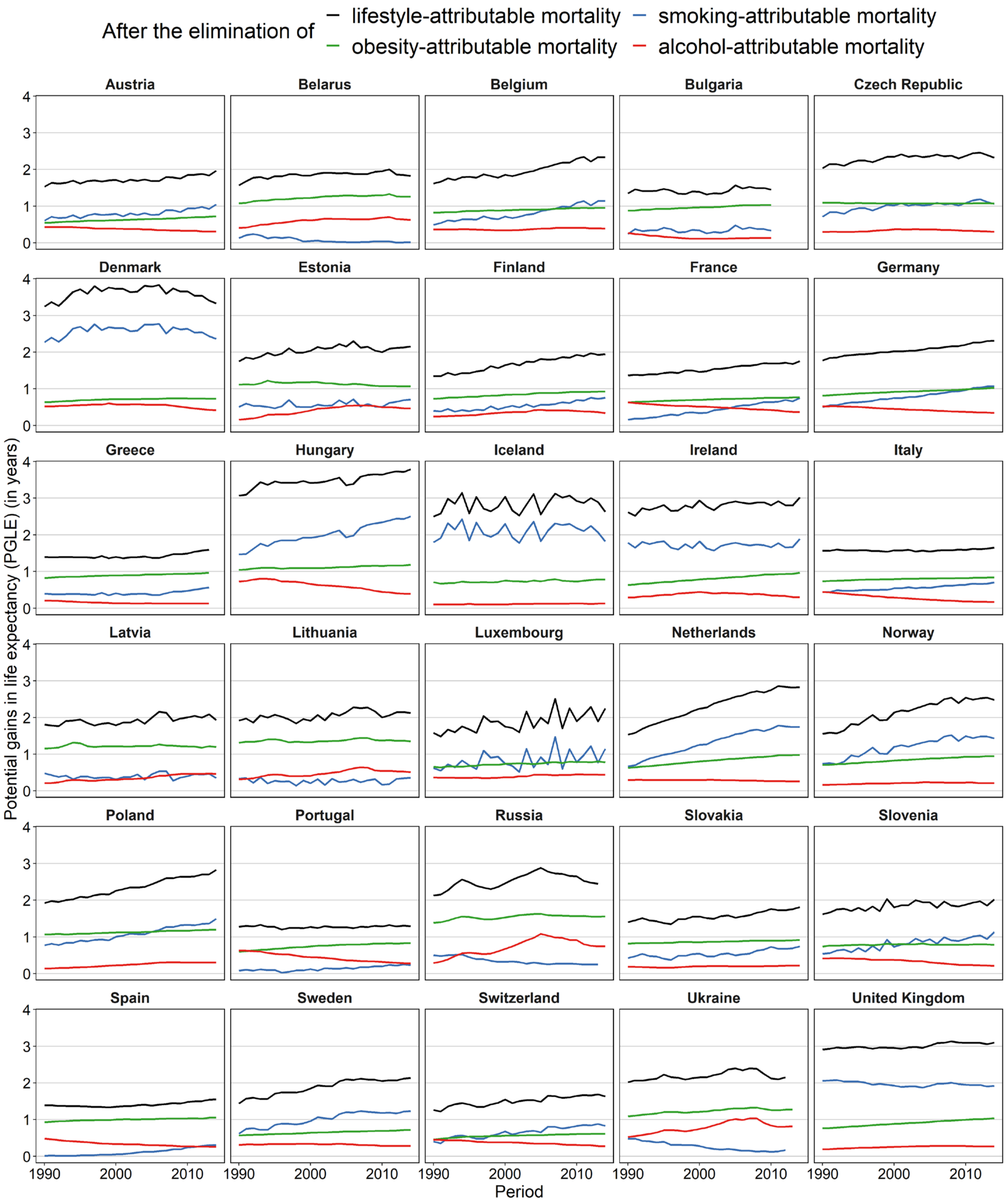
After the elimination of
lifestyle-attributable mortality
smoking-attributable mortality
obesity-attributable mortality
alcohol-attributable mortality
Austria
Belarus
Belgium
Bulgaria
Czech Republic
Denmark
Estonia
Finland
France
Germany
Greece
Hungary
Iceland
Ireland
Italy
Latvia
Lithuania
Luxembourg
Netherlands
Norway
Poland
Portugal
Russia
Slovakia
Slovenia
Spain
Sweden
Switzerland
Ukraine
United Kingdom
Potential gains in life expectancy (PGLE) (in years)
0
1
2
3
4
1990
2000
2010
Period

Supplementary Figure S3a - Trends in life expectancy at birth (e0), observed (= all-cause mortality) versus after excluding smoking-, obesity- and alcohol-attributable mortality (separately and combined), 1950-2014*, by country, men. * Based on the available information**.** Lifestyle refers to smoking, obesity and alcohol combined.

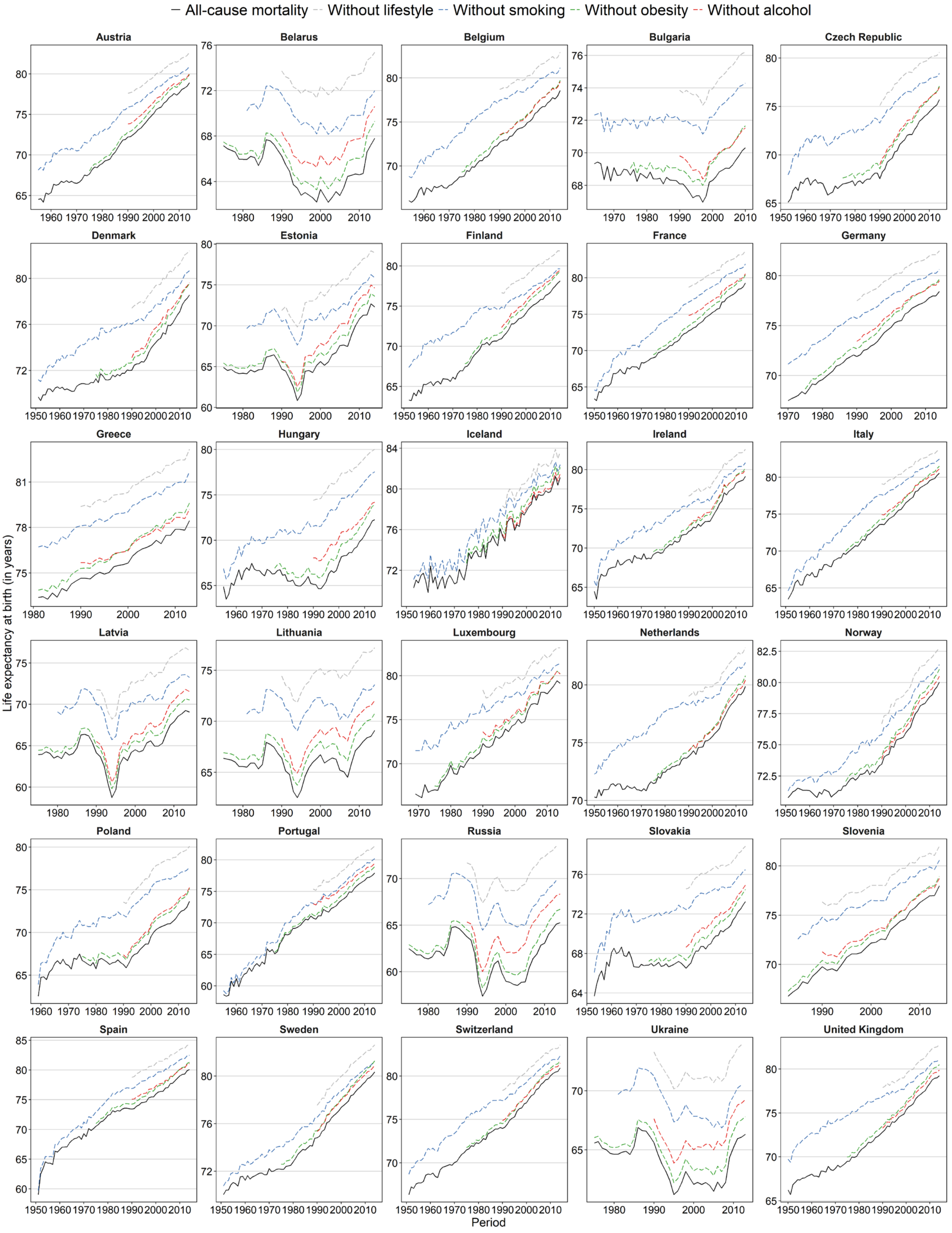

Supplementary Figure S3b - Trends in life expectancy at birth (e0), observed (= all-cause mortality) versus after excluding smoking-, obesity- and alcohol-attributable mortality (separately and combined), 1950-2014*, by country, women. * Based on the available information. Lifestyle refers to smoking, obesity and alcohol combined.

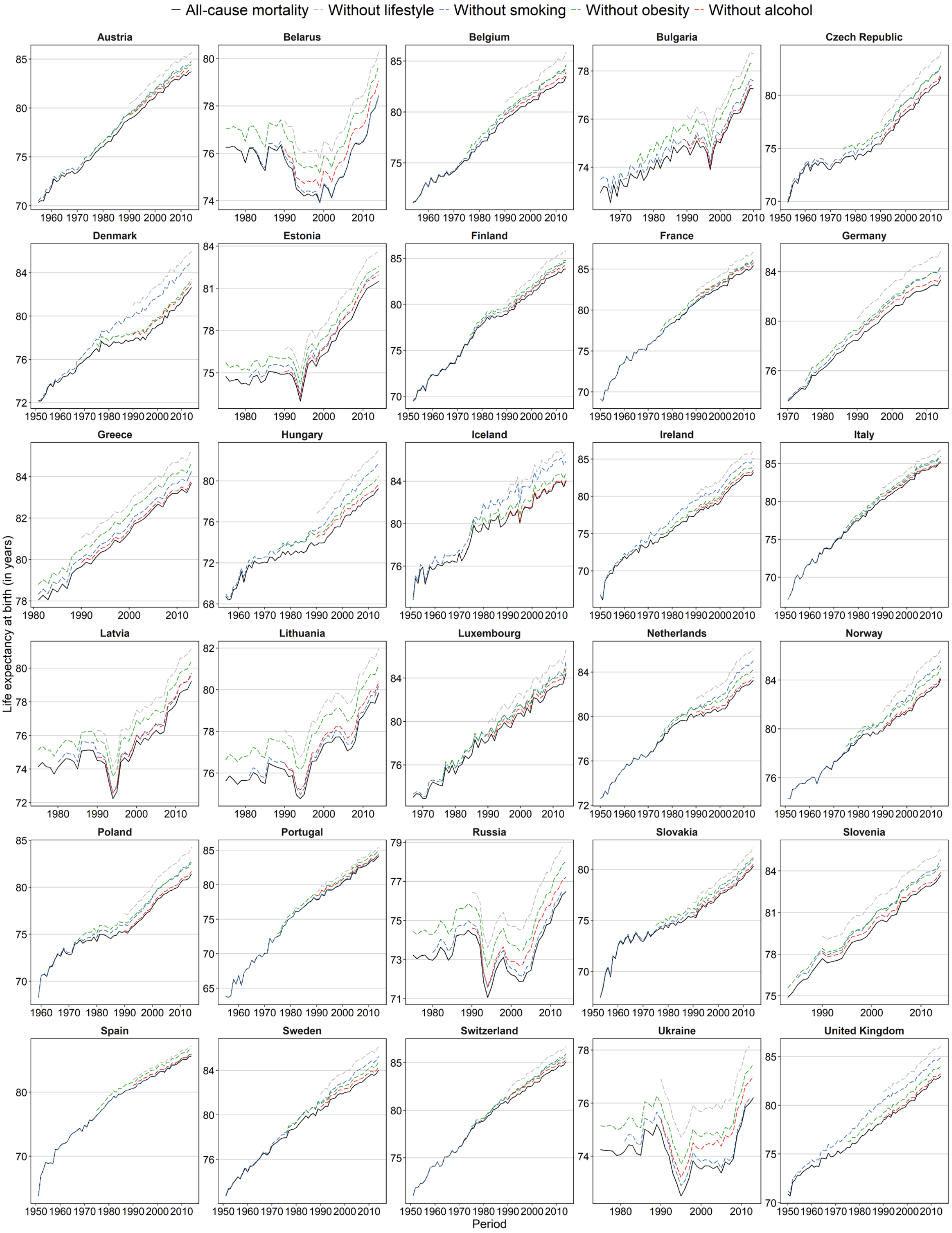

Supplement: dyaa273_Supplementary_Data [file dyaa273_supplementary_data.zip › ije-2020-05-0940-File003.pdf]
